# Effectiveness of seasonal influenza vaccinations against laboratory-confirmed influenza-associated infections among Singapore military personnel in 2010–2013

**DOI:** 10.1111/irv.12256

**Published:** 2014-05-14

**Authors:** Hin Peow Ho, Xiahong Zhao, Junxiong Pang, Mark I-C Chen, Vernon J M Lee, Li Wei Ang, Raymond V Tzer Pin Lin, Christine Q Gao, Li Yang Hsu, Alex R Cook

**Affiliations:** a Biodefence Centre, Ministry of DefenceSingapore; b Centre for Infectious Disease Epidemiology and Research, Saw Swee Hock School of Public Health, National University of SingaporeSingapore; c National University Health SystemSingapore; d Department of Clinical Epidemiology, Tan Tock Seng HospitalSingapore; e Epidemiology & Disease Control Division, Ministry of HealthSingapore; f National Public Health Laboratory, Ministry of HealthSingapore; g Yale-NUS College, National University of SingaporeSingapore

**Keywords:** influenza A(H1N1)pdm09, influenza A(H3N2), influenza B, influenza vaccination, vaccine effectiveness

## Abstract

**Background:**

Limited information is available about seasonal influenza vaccine effectiveness (VE) in tropical communities.

**Objectives:**

Virus subtype-specific VE was determined for all military service personnel in the recruit camp and three other non-recruit camp in Singapore's Armed Forces from 1 June 2009 to 30 June 2012.

**Methods:**

Consenting servicemen underwent nasal washes, which were tested with RT-PCR and subtyped. The test positive case and test negative control design was used to estimate the VE. To estimate the overall effect of the programme on new recruits, we used an ecological time series approach.

**Results:**

A total of 7016 consultations were collected. The crude estimates for the VE of the triavalent vaccine against both influenza A(H1N1)pdm09 and influenza B were 84% (95% CI 78–88%, 79–86%, respectively). Vaccine efficacy against influenza A(H3N2) was markedly lower (VE 33%, 95% CI −4% to 57%). An estimated 70% (RR = 0·30; 95% CI 0·11–0·84), 39% (RR = 0·61;0·25–1·43) and 75% (RR = 0·25; 95% CI 0·11–0·50) reduction in the risk of influenza A(H1N1)pdm09, influenza A(H3N2) and influenza B infections, respectively, in the recruit camp during the post-vaccination period compared with during the pre-vaccination period was observed.

**Conclusions:**

Overall, the blanket influenza vaccine programme in Singapore's Armed Forces has had a moderate to high degree of protection against influenza A(H1N1)pdm09 and influenza B, but not against influenza A(H3N2). Blanket influenza vaccination is recommended for all military personnel.

## Introduction

Singapore is a city state that lies off the southern tip of the Malay Peninsula. Its location, <200 km from the equator, bestows a near constant year-round temperature and humidity and only moderately elevated rainfall during the monsoons. As a result, the timing of influenza outbreaks in Singapore is much less predictable than in temperate countries, with year-round potential for influenza transmission.

All male residents of Singapore undertake 2 years of national service on reaching adulthood and most serve in the Singapore Armed Forces (SAF). Servicemen initially undertake training at a dedicated camp (‘the recruit camp’ henceforth) off the main island of Singapore, Pulau Ujong. On completing training, they are posted to other camps, mostly on Pulau Ujong itself. Their service takes place in a semi-closed environment, and servicemen usually return to their families during the weekend, which may promote the transmission of infections between servicemen and the public. While on camp, soldiers live, work and socialise in close proximity, with potential for large outbreaks of respiratory infections such as influenza, akin to those observed in other closed or semi-closed populations, such as boarding schools or prisons.^1^

The SAF implemented routine vaccination of new recruits with the H1N1-2009 vaccine when it became available at the end of 2009. Previous research found that this policy reduced H1N1-2009 disease burden among servicemen and hence recommended routine influenza vaccination of new recruits with the trivalent vaccine.^2^ Other settings have seen mixed findings on the effectiveness of non-pandemic or seasonal influenza vaccines. A study of seasonal influenza vaccine effectiveness (VE) among US military basic trainees in the 2005–2006 season showed that the VE against laboratory-confirmed influenza was 92%.^3^ Outside militaries, a 2011 systemic review showed pooled effectiveness of 59% for seasonal influenza by trivalent inactivated vaccine in the protection of adults aged 18–65 years.^4^ Other post-pandemic studies revealed an overall VE of 56% in the UK,^5^ 60% in the USA,^6^ 55% in Spain,^7^ and 30% against severe cases in high-risk individuals in France for H1N1-2009.^8^ Although these estimates indicate only moderate effectiveness on an individual basis, the protection afforded by herd immunity in a closed or semi-closed population, such as the SAF, may potentially result in greater effectiveness for a vaccine programme in reducing morbidity and absenteeism.

There is a paucity of studies that assess the effectiveness of blanket seasonal influenza vaccination over time in the same community. As a result, the expansion of the SAF vaccination programme to include all servicemen using the trivalent vaccine from 2011 onwards provides a unique opportunity to assess the effect of mass influenza vaccination to approximately half of an entire birth year cohort in a country. We estimated effectiveness of the 2011–2012 and 2012–2013 trivalent influenza vaccines in the Singapore military population using the standard test positive and test negative methodology, and the impact of the programme on reducing both influenza-related morbidity and overall febrile respiratory illness using a time series analysis approach.

## Methods

### Subjects recruitment

The SAF have maintained a respiratory illness surveillance programme in four sentinel camps, including the recruit camp, since 2009. The recruit camp is the primary focus for analysis because all servicemen spend the first 3 months of their service there, after which many will be assigned to camps not in the surveillance network. The three other camps in the sentinel network are also considered. The surveillance population of interest in this study is all military service personnel in these four camps from 1 June 2009 to 30 June 2012. These comprise recruits who are medically fit and age 19 years at enlistment undergoing training, as well as permanent staff stationed there, such as trainers, cooks and medical officers. The camp has a medical facility, which operates 24 hours daily.

The inclusion criteria for the study are all military personnel who visited the camp medical facility with a fever >37·5°C and a cough or sore throat or both, who are clinically diagnosed as having a Febrile Respiratory Illness (FRI) and who were within 72 hours of illness onset. Servicemen meeting these criteria had a nasal wash specimen collected, which was placed in viral transport media and sent to the laboratory for further analysis within 24 hours.^9^ A temperature cut-off of 37·5°C was selected as its sensitivity of 82% for seasonal influenza is higher than the elevated thresholds of 37·8°C and 38·0°C (sensitivity of 74% and 63%, respectively).^10^ Subjects with repeat visits for the same illness episode had only the first visit included for the study.

### Vaccination programme

The trivalent seasonal influenza vaccination was first introduced to the recruit camp in late 2010, followed by all other camps in the Singapore military in late 2011. Thereafter, all personnel in the camps have received the prevailing trivalent seasonal vaccine, where vaccine formulations are based on the recommendations of the World Health Organization (WHO) for each northern and southern hemisphere influenza season.^11^ Prior to this study, seasonal influenza vaccination was given routinely only to healthcare workers and select military personnel in critical vocations.

From 01 December 2009 to 31 October 2010, the monovalent pandemic H1N1 vaccine was introduced to recruits in the recruit camp. This was superseded by trivalent vaccines which were later extended to non-recruits (Tables S1 and S2). New recruits were vaccinated shortly upon enlistment, which occurred throughout the year, whereas servicemen in other military camps were vaccinated in advance of the north/south influenza seasons.^12^ Vaccine composition is detailed in Table S3. To facilitate the analysis, the study is subdivided into four time periods. In period 1, from 31 May 2009 to 30 November 2009, no vaccination was given. In period 2, from 1 December 2009 to 31 October 2010, new recruits were given the monovalent pandemic H1N1 vaccine. In period 3, from 1 November 2010 to 30 September 2011, recruits from the recruit camp were given the trivalent vaccine. In period 4, from 1 October 2011 to 30 June 2012, servicemen from all the military camps, including the recruit camp, were given the trivalent vaccine.

### Laboratory testing

Influenza viral detection (influenza A and B) was carried out using ResPlex II (Qiagen, Singapore, Singapore), modified to include testing for influenza A(H1N1)pdm09. Subtyping of samples detected as influenza A positive was carried out using real-time reverse transcription PCR. Samples tested positive for influenza A, but not A(H1N1)pdm09 by ResPlex II were subjected to another round of testing with real-time PCR for subtyping. In total, 7016 samples were tested.

### National data on influenza and upper respiratory tract infections

The Ministry of Health (MOH), Singapore, has a national surveillance programme for influenza, which monitors attendances for upper respiratory tract infections (URTI) at government clinics. Under its sentinel surveillance programme, nasopharyngeal, nasal and/or throat swabs are taken from patients with influenza-like illness (ILI) (temperature >38°C with either cough or sore throat) at government primary care clinics and private general practitioner clinics for influenza subtyping.

### Statistical analysis

Exposure is determined by the vaccine history of cases with FRI and controls: those never vaccinated, or vaccinated within the last 14 days, were treated as unexposed, while those vaccinated more than 14 days ago were treated as exposed. Being ‘vaccinated’ was determined by the virus and time period: monovalent or trivalent vaccines for FRI and influenza A(H1N1)pdm09 and trivalent for influenza A(H3N2) and influenza B.

### Test positive and test negative design

The test positive case and test negative control design^13^ was used to estimate the VE of the monovalent vaccine, as well as the trivalent vaccine on various influenza virus subtypes [influenza A(H1N1)pdm09, influenza A(H3N2) and influenza B] across all four surveillance camps. Servicemen with FRI whose nasal wash specimens tested positive against the influenza virus subtypes were defined as cases, while those with FRI who tested negative against the influenza virus subtypes were defined as controls. Both crude and adjusted VE were estimated in this article, where the adjusted VE was calculated as 100% × (1−adjusted odds ratio) using logistic regression. Variables adjusted for were camp groups (recruit camp versus other camps), vaccination periods, individual vaccination history (received or not), as well as the interaction between individual vaccination history and camp groups. To account for the delay from vaccination to protection,^14^ we repeated the analysis treating servicemen who have been vaccinated for ≤14 days from symptom onset as unvaccinated. The model presented was obtained by dropping terms from the saturated model which were not statistically significant at the 0·05 level. Potential confounders included in the saturated model are age, gender, camp groups, vaccination periods, individual vaccination history and an interaction term between individual vaccination history and camp groups. If the 95% confidence interval (CI) for the VE for any subtype included zero, we concluded there was insufficient evidence of effectiveness against that virus subtype.

### Ecological time series analysis

To estimate the overall effect of the programme on new recruits, we used an ecological time series approach. Non-recruit camps were omitted due to their diverse vaccination background, as servicemen vaccinated as recruits and in other camps are frequently re-assigned to these camps. An autoregressive generalised linear model (quasi-Poisson with the log-link function) was built to determine the possible association between monovalent pandemic or trivalent seasonal influenza vaccination and influenza cases developing post-vaccination. The number of cases in the recruit camp for the previous week (separately for before and after the vaccination programme) and the number of cases detected by the national surveillance programme were accounted for in the model to remove the confounding effect of infections acquired by the subjects from outbreaks in the community. In particular, the inclusion of the circulation of each influenza virus subtype in the community allowed changes to the dominant circulating subtype to be accounted for. To obtain the final model, covariates were dropped if they were not statistically significant at the 0·05 level. The relative risk estimate and its corresponding 95% CI for each term were provided.

Pearson's chi-square test was used to examine differences in proportions within groups. Data used for all analyses were pooled across study periods from 31 May 2009 to 30 June 2012. Analyses were performed using the R Statistical Software (version 2.15.2).^15^

Written informed consent was obtained from the study participants. The study was approved by the SAF's Joint Medical Committee for Research, and the institutional review board of the National University of Singapore.

## Results

There were 7016 FRI consultations between 31 May 2009 and 30 June 2012 across the four camps in the surveillance network, tabulated by subtype in Table S4. The mean age of participants was 22·7 years with a range of 18–62 years, and the sex distribution was overwhelmingly male (99·8%, 7000 of 7016, see Table1). A majority (71·8%, 5036 of 7016) of the FRI cases were from the recruit camp. In all, 20·2% (1415 of 7016) had received the monovalent pandemic H1N1 vaccination, and 36·9% (2589 of 7016) the trivalent seasonal influenza vaccination. Of these cases, 7·3% (513), 1·4% (99) and 8·4% (586) tested positive for influenza A(H1N1)pdm09, influenza A(H3N2) and influenza B, respectively. Individuals in the 25–62 years age group were more likely to be positive for influenza A(H1N1)pdm09 (12·2%), while younger participants were more likely to be positive for influenza B than the other influenza types (9·0%). There was significant heterogeneity in the age distribution within each respective subtype grouping of influenza cases (χ^2^ = 48·4, 6·9, 21·8; *P* = 0·0005, 0·04, 0·0005, respectively).

**Table 1:** Descriptive characteristic of Singapore Armed Forces (SAF) servicemen with febrile respiratory illness (FRI) by age group, gender, camp, time period, vaccination status and subtype, 31 May 2009 to 30 June 2012 (*n* = 7016)

Subjects characteristic	No. of samples	Influenza A(H1N1)pdm09		Influenza A(H3N2)		Influenza B	
		
		No. of Positives (%)	*P*-value*	No. of Positives (%)	*P*-value*	No. of Positives (%)	*P*-value*
Age							
15–19	326	3 (0·9)	<0·001	1 (0·3)	0·04	18 (5·5)	<0·001
20–24	5884	412 (7·0)		80 (1·4)		531 (9·0)	
25–62	806	98 (12·2)		18 (2·2)		37 (4·6)	
Gender							
Male	7000	512 (7·3)	1	99 (1·4)	1	585 (8·4)	
Female	16	1 (6·3)		0 (0·0)		1 (6·3)	
Camp							
Recruit Camp	5036	359 (7·1)	0·36	50 (1·0)	<0·001	511 (10·1)	<0·001
Other camps	1980	154 (7·8)		49 (2·5)		75 (3·8)	
Time period							
31 May 2009–30 November 2009	1336	294 (22·0)	<0·001	12 (0·9)	0·14	4 (0·3)	<0·001
1 December 2009–31 October 2010	2431	156 (6·4)		44 (1·8)		480 (19·7)	
1 November 2010–30 September 2011	1784	56 (3·1)		23 (1·3)		42 (2·4)	
1 October 2011–30 June 2012	1465	7 (0·5)		20 (1·4)		60 (4·1)	
Monovalent vaccination							
Yes	1415	43 (3·0)	<0·001	24 (1·7)	0·31	311 (22·0)	<0·001
No	5601	470 (8·4)		75 (1·3)		275(4·9)	
Trivalent vaccination							
Yes	2589	49 (1·9)	<0·001	28 (1·1)	0·08	56 (2·2)	<0·001
No	4427	464 (10·5)		71 (1·6)		530 (12·0)	

* By Pearson's chi-square test, comparing proportions within groups.

Within the recruit camp, the most common causative agent isolated was influenza B (10·2%), followed by influenza A(H1N1)pdm09 (7·1%). In other camps, influenza A(H1N1)pdm09 was the most frequent causative agent isolated (7·8%), followed by influenza B (3·8%). No between-camp heterogeneity was observed for influenza A(H1N1)pdm09 isolates (χ^2^ = 0·9; *P* = 0·36). With each succeeding time period for vaccination coinciding with the expansion of the vaccination programme, there was a significant decrease in the proportion of cases positive for influenza A(H1N1)pdm09 (χ^2^ = 575·3; *P* < 0·001); there was no such trend for influenza A(H3N2) and influenza B. Although the proportions testing positive were also significantly different by time periods, this was only statistically significant for influenza A(H1N1)pdm09 and influenza B (χ^2^ = 643·9; *P* < 0·001). Subjects who had received the monovalent pandemic H1N1 vaccine had a significantly lower proportion of influenza A(H1N1)pdm09 isolates (3·0%) than those who did not (8·4%; χ^2^ = 47·8; *P* < 0·001), but had a higher proportion of influenza B isolates (22%) than those who did not (4·9%, χ^2^ = 429·9; *P* < 0·001). Subjects who had received the trivalent vaccine had significantly smaller proportions of influenza A(H1N1)pdm09 (1·9% versus 10·5% in unvaccinated; χ^2^ = 142·8; *P* < 0·001) and influenza B isolates (2·2% versus 12·0%; χ^2^ = 205·3; *P* < 0·001), but no statistically significant difference in the proportions testing positive for influenza A(H3N2) by vaccination status (1·1% versus 1·6%; χ^2^ = 3·2; *P* = 0·08) was observed.

### Influenza time series analysis

**Figure 1:**
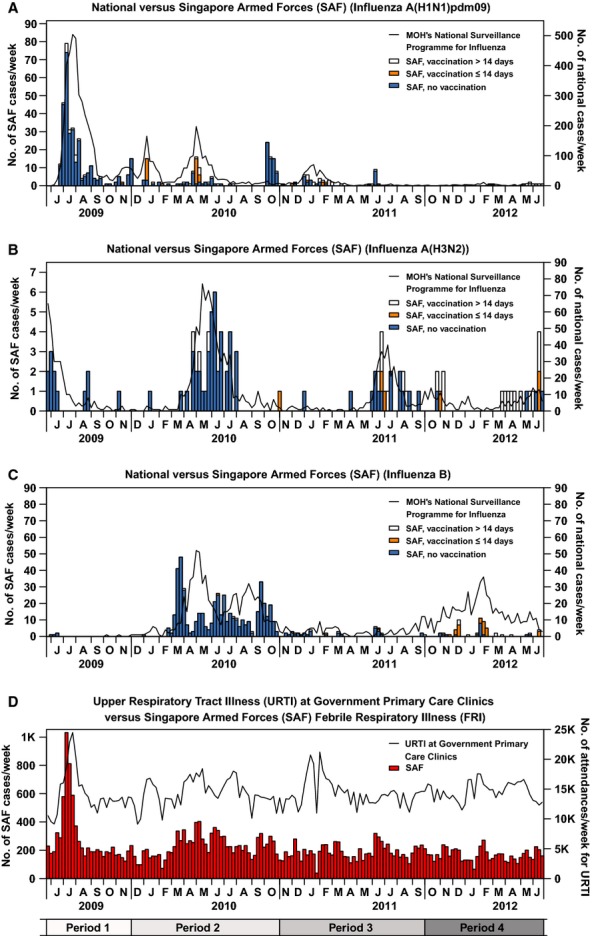
Distribution of Weekly National Influenza Cases versus Singapore Armed Forces (SAF) Influenza Cases by Subtype (A, B, C) and Weekly National Upper Respiratory Tract Infection (URTI) Cases versus SAF Febrile Respiratory Illness (FRI) Cases (D). On panels A–C, national influenza cases are represented by lines, while SAF influenza cases are represented by bars (blue for unvaccinated, orange for those vaccinated ≤14 days prior to consultation and white for vaccinated >14 days prior). On panel D, weekly national URTI cases are represented by the black line, while the weekly FRI cases in military camps are represented by red bars. The scales for *y*-axes used are different for B and D. The time is measured in calendar months on each panel, and the longer tick marks at each *x*-axis represent the start and end of each vaccination period. An additional time axis is presented at the foot, where period 1 refers to the pre-vaccination period; period 2 refers to the period new recruits were given monovalent vaccination; period 3 refers to the period new recruits were given trivalent vaccination; and period 4 refers to the period all SAF servicemen received trivalent vaccination.

The time series data for the military and national surveillance of laboratory-confirmed influenza cases are presented in Figure1. For influenza A(H1N1)pdm09 in Period 1 when there was no vaccination programme, and influenza B in Period 2 when recruits were given monovalent vaccine, the epidemic peaks for the military cohort often preceded that for the national cohort by 2–3 weeks. The influenza epidemic peaks in the military cohort generally coincide with enlistment of new recruits into the recruit camp, which occurs mostly during the periods of February–March, May–June and September–October.

Influenza A(H1N1)pdm09 activity peaked in July 2009, although there were also several subsequent smaller peaks of activity in Periods 2 and 3 when recruits were receiving monovalent and then trivalent vaccination, but almost no activity in period 4 when trivalent vaccination was fully rolled out. Since March 2010, there was resurgent epidemic activity of influenza A(H3N2) and influenza B. Influenza B epidemic activity in the SAF was subsequently attenuated with smaller peaks observed in the periods of June–July 2011 and February–March 2012, respectively, in spite of considerably high levels of activity based on the data from MOH's national surveillance programme for influenza. In the military cohort for all the influenza subtypes, following the roll-out of vaccination programmes from Period 2 onwards, there was a notably large proportion of laboratory-confirmed cases who had onset within 2 weeks of vaccination.

Smaller peaks in FRI cases/week from period 2 to period 4 followed a sharp spike in period 1, due to the first wave of the H1N1 pandemic. Subsequent epidemic peaks become more attenuated over time. These patterns were replicated in the national surveillance system, but without the attenuation over time.

### Vaccine effectiveness

Table2 shows that for the monovalent vaccine, the crude estimate for the VE against influenza A(H1N1)pdm09 was 66% (95% CI 53–75%) for the analysis in which all subjects who had been vaccinated were classified in the vaccination group. For the trivalent vaccine, the crude estimates for the VE against influenza A(H1N1)pdm09 and influenza B were both 84% (95% CI 78–88%, 79–86%, respectively). The crude VE of the trivalent vaccine against influenza A(H3N2) was markedly lower (VE 33%, 95% CI −4% to 57%). When adjusted for camp groups, the monovalent vaccine revealed statistical evidence of an effect (VE 60%, 95% CI 2–88%). However, when adjusted for camp groups, the VE of the trivalent vaccine against influenza A(H1N1)pdm09 and influenza A(H3N2) did not (VE −34%, 9%, 95% CI −99% to 12%, −79% to 55%, respectively), and the VE against influenza B dropped to 61% (95% CI 25–81%).

**Table 2:** Crude and adjusted influenza monovalent and trivalent vaccine effectiveness by camp and subtype, 31 May 2009 to 30 June 2012 (*n* = 7016)

Subtype	Influenza vaccine effectiveness (%) [95% confidence interval (CI)]					

	Subjects with ≤14 days vaccination being classified as vaccination group (*n* = 7016)			Subjects with ≤14 days vaccination being classified as no vaccination group (*n* = 7016)		
	
	Crude	Adjusted*		Crude	Adjusted*	
	
		Recruit Camp	Other Camps		Recruit Camp	Other Camps
Monovalent Vaccine effectiveness						
Influenza A(H1N1)pdm09, Period 2**	72 (59–81)	80 (70–87)	65 (−48 to 92)	94 (87–98)	97 (92–99)	63 (−57 to 91)
Trivalent Vaccine effectiveness						
Influenza A(H1N1)pdm09, Period 3**	71 (49–84)	81 (64–90)	5 (−222 to 72)	69 (41–84)	76 (49–88)	30 (−162 to 81)
Influenza A(H1N1)pdm09, Period 4**	83 (23–96)	97 (49–100)	44 (−208 to 90)	46 (−144 to 88)	89 (−66 to 99)	24 (−331 to 87)
Influenza A(H3N2), Period 3**	60 (7–83)	−115 (−1748 to 75)	42 (−80 to 82)	59 (−5 to 84)	1 (−391 to 80)	56 (−56 to 88)
Influenza A(H3N2), Period 4**	49 (−55 to 83)	80 (16–95)	−73 (−127 to 78)	24 (−91 to 70)	71 (−23 to 93)	3 (−357 to 79)
Influenza B, Period 3**	86 (69–93)	90 (77–96)	69 (−163 to 96)	88 (66–96)	91 (71–97)	65 (−192 to 96)
Influenza B, Period 4**	70 (45–83)	71 (45–85)	65 (−39 to 91)	92 (84–96)	95 (88–98)	72 (−1·8 to 92)
Overall Influenza***, Period 3**	77 (65–84)	83 (73–90)	39 (−34 to 72)	76 (61–85)	81 (66–89)	52 (−13 to 79)
Overall Influenza***, Period 4**	69 (49–81)	77 (59–87)	34 (−83 to 76)	83 (73–89)	93 (86–97)	47 (−31 to 79)

* Variables being adjusted in all logistic regression models are camp group, vaccination period, vaccination history and interaction between vaccination history and camp group.

** Period 1 refers to the pre-vaccination period; period 2 refers to the period new recruits were given monovalent vaccination; period 3 refers to the period new recruits were given trivalent vaccination; and period 4 refers to the period all SAF servicemen received trivalent vaccination.

*** The overall influenza includes influenza A(H1N1)pdm09, influenza A(H3N2) and influenza B.

For the analysis in which only subjects more than 14 days from the day of vaccination were classified in the vaccinated group, the crude VE of the monovalent vaccine against influenza A(H1N1)pdm09 was higher at 88% (95% CI 79–94%). For the same analysis, the crude VE of the trivalent vaccine against influenza A(H1N1)pdm09 was marginally lower at 80% (95% CI 72–85%), and the crude VE against influenza B was higher at 93% (95% CI 88–95%). There was no statistically discernible protection of the trivalent vaccine against influenza A(H3N2), and at best moderate protection (VE 31%, 95% CI −11% to 57%). When adjusted for camp groups, the VE of the monovalent vaccine against influenza A(H1N1)pdm09 in the recruit camp was higher than in the first analysis (VE 89%, 95% CI 77–94%). The VE of the trivalent vaccine against influenza A(H1N1)pdm09 in the recruit camp was marginally lower than in the first analysis (VE 78%, 95% CI 56–89%), and the VE against influenza B in the recruit camp was higher than in the first analysis (VE 94%, 95% CI 88–97%). The VE of the monovalent vaccine against influenza A(H1N1)pdm09 at 59% (95% CI 1–88%) was lower than that in the recruit camp. The VE of the trivalent vaccine was not statistically significant for influenza A(H1N1)pdm09 and influenza A(H3N2) in the other camps (VE −41%, 95% CI −111% to 8% and VE 12%, 95% CI −71% to 57%, respectively). The VE of the trivalent vaccine at 58% (95% CI 19–80%) against influenza B in the other camps was lower than that in the recruit camp. The classification of subjects with more than 14 days from date of vaccination in the vaccination group did not alter the general direction of the VE in the various subgroups. VE estimates were robust when stratified by time period (Table S5).

From the ecological time series analysis, it was observed that there were estimated reductions of 70% (RR = 0·30; 95% CI 0·11–0·84), 39% (RR = 0·61;0·25–1·43) and 75% (RR = 0·25; 95% CI 0·11–0·50) in the risk of influenza A(H1N1)pdm09, influenza A(H3N2) and influenza B infections, respectively, in the recruit camp during the post-vaccination period compared with during the pre-vaccination period (Table3). In general, there was a lower baseline infection rate in the period when vaccination was performed, particularly for influenza A(H1N1)pdm09 and influenza B infections. There was, however, no discernible reduction on overall FRI cases as a result of the vaccination programme (Table3).

**Table 3:** Relative risks for influenza infection from the ecological time series analysis, 31 May 2009 to 30 June 2012. Potential confounders included in all models are vaccination period, the number of cases in the recruit camp for the previous week before and after the vaccination programme and positive samples detected by the national surveillance programme

Model	Relative Risk [95% Confidence Interval (CI)]				No. of vaccinated positive cases in recruits during vaccination period (%)	
	
	Vaccination Period versus No Vaccination Period*	AR Term in No Vaccination Period	AR Term in Vaccination Period	NPHL positive samples** (per 10 positive samples)	Subjects with ≤14 days vaccination being classified as vaccination group	Subjects with ≤14 days vaccination being classified as no vaccination group
Influenza A(H1N1)pdm09	0·30 (0·11–0·84)	1·01 (0·99–1·03)	1·16 (1·09–1·24)	1·05 (1·02–1·08)	53 (32·3)	17 (10·4)
Influenza A(H3N2)	0·61 (0·25–1·43)	1·29 (0·96–1·71)	2·25 (1·02–4·48)	1·45 (1·27–1·65)	10 (71·4)	6 (42·9)
Influenza B	0·25 (0·11–0·50)	1·06 (1·05–1·07)	1·19 (1·03–1·34)	1·31 (1·10–1·53)	43 (50·6)	8 (9·4)
FRI	0·84 (0·60–1·19)	1·00 (1·00–1·01)	1·01 (1·00–1·01)	1·01 (1·00–1·02)	3183 (75·2)	2390 (56·5)

AR, Autoregressive; NPHL, National public health laboratory; FRI, Febrile respiratory illness.

* No vaccination periods for each response variable are as follows: 31 May 2009 to 30 November 2009 for FRI and influenza A(H1N1)pdm09, 31 May 2009 to 31 October 2010 for influenza A(H3N2) and influenza B.

** NPHL data used for each response variable are as follows: total NPHL positive cases for FRI, A(H1N1)pdm09 NPHL positive cases for influenza A(H1N1)pdm09, A(H3N2) NPHL positive cases for influenza A(H3N2) and B NPHL positive cases for influenza B.

**Figure 2:**
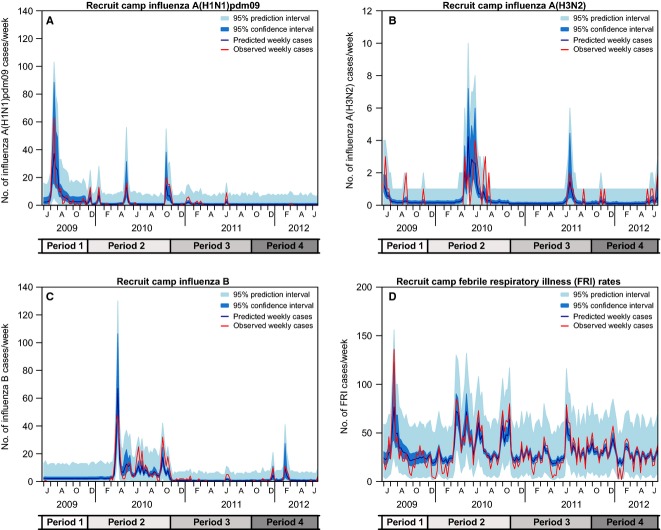
Comparison of Recruit Camp Influenza Cases (A, B, C) and Febrile Respiratory Illness (FRI) Cases (D) with Predictions from the Ecological Time Series Model. Lines represent observations (red) and predictions (blue). Shaded regions are 95% confidence intervals (dark blue) and prediction intervals (light blue). The *y*-axis scales differ for B and D.

There was good concordance between the fitted model and observed numbers of cases of influenza by subtype, or FRI, in the recruit camp (Figure2).

To investigate the association between VE and the number of vaccinations received, a stratified analysis was conducted. Significantly lower VEs were observed for those receiving more than one vaccination for the monovalent VE against influenza A(H1N1)pdm09 (*P*-value: <0·001) and the trivalent VEs against influenza A(H1N1)pdm09 and influenza A(H3N2) (*P*-value: <0·001 and <0·001 respectively), but not for the trivalent VE against influenza B (*P*-value: 0·74).

## Discussion

The blanket influenza vaccine programme rolled out in SAF has had a moderate to high degree of protection against influenza A(H1N1)pdm09 and influenza B, but not against influenza A(H3N2) that circulated in SAF during the programme's roll-out. The VE estimates for the recruit camp were substantially higher than in a US military population in the 2010–2011 season and those reported for the early 2011–2012 season in the European Union.^16^

It is not clear why we observed a low VE against influenza A(H3N2). Other researchers who have had a low VE for this virus have postulated a vaccine mismatch against the circulating strain of influenza A(H3N2).^17^ For influenza A(H1N1)pdm09 and influenza B viruses, drift variants often co-circulate with multiple co-existing lineages, allowing the re-emergence of old strains. In contrast, influenza A(H3N2) subtype viruses undergo antigenic drift much more frequently and new variants often replace old ones.^18^ In preliminary analysis, we found the VE fell as the period after vaccination in which soldiers were counted as unvaccinated lengthened (data not shown), suggesting that the lower VE in non-recruit camps may be explained by the observation that as vaccination programmes were initially rolled out only in recruit camps, many individuals in other camps who received vaccination as recruits would have had a longer post-vaccination-to-infection period than vaccinated soldiers in the recruit camp. Hence, the reduced VE could be due to the waning of antibody levels following vaccination, which has been described in several other studies.^19^ In a separate analysis, in which the VEs estimates were stratified by the number of vaccinations received, there was a statistically significantly reduced VE for those with multiple vaccinations (for all except influenza B). This may reflect the waning of the protective effect of vaccination, although a meta-analysis of serologic and field studies published between 1966 and 1997 did not detect any evidence for a decreasing protection with annually repeated influenza vaccination, so this finding may be an artefact of the study design.^20^

National surveillance of URTI cases indicate continued presence of outbreaks in the community during periods 2–4 (Figure1), in contrast to the military FRI cases/week, which can be explained by at-risk groups being under-vaccinated relative to international recommendations.^21^

The resurgent influenza A(H3N2) and influenza B activity in the SAF observed in the early phase of the vaccination programme (period 2, Figure1), when recruits were receiving monovalent vaccine against influenza A(H1N1)pdm09, was paralleled in the general population. However, there was reduced influenza B epidemic activity in the SAF after 2010, despite the large outbreak in the community in 2011–2012. The time series analyses we presented corroborated results from the test positive and test negative study design that the vaccine was effective against influenza A(H1N1)pdm09 and influenza B. In addition, the large reductions in relative risk of influenza A(H1N1)pdm09 and influenza B following introduction of the monovalent and trivalent vaccines, respectively, provide evidence of the success of our influenza vaccination programme in reducing morbidity rates associated with influenza infection and the concomitant loss of training days, with very few servicemen testing positive for these two subtypes after programme implementation. Moreover, of those servicemen testing positive in later periods, a substantial 46% received their vaccination more than 2 weeks ago prior before their onset of symptoms, that is, during the period before vaccine-induced influenza antibody titres peak.^22^ Despite the consistent implementation of the trivalent influenza vaccination exercise in the military cohort, due to the high turnover of recruits in the camp, there are still small outbreaks of epidemic activity in new enlistees who have not been vaccinated yet or whose antibodies have not yet responded to vaccination. Potentially, these outbreaks could be prevented by vaccinating recruits shortly before they enter the camp.

An apparently harmful effect of monovalent vaccination on influenza B, as reflected by the higher percentage of influenza B positive samples in servicemen who had received monovalent vaccination, compared with those who had not, was observed (Table1, 22% versus 4·9%). This did not survive adjustment for confounders and should therefore be considered spurious.

There are a few limitations to this study. Firstly, our study is limited to febrile presentations of influenza and may not be applicable to milder presentation of influenza. Secondly, while we demonstrated overall reduction in burden of disease from influenza, there was almost no reduction in overall FRI case counts (Figure1) after the roll-out of the vaccination programme. We are uncertain whether there was some degree of replacement of influenza infections by adenovirus and other febrile respiratory pathogens, particularly because we have observed in testing FRI cases for other viruses (data not shown) an absolute increase in the burden of disease attributable to adenovirus following the implementation of SAF-wide influenza vaccination. Further assessment of such an effect would require that we control for the circulating incidence of such pathogens in the community over the same time periods (as was done in our time series analysis for influenza). Finally, our estimates of vaccine effectiveness cannot be generalised to the general population, who are more heterogeneous than national servicemen, or even to the military in other countries in which the structure of army life may differ.

In conclusion, the mass influenza vaccination programme started in the Singapore military in 2009 substantially reduced influenza infections. It was effective against influenza A(H1N1)pdm09 and influenza B, but offered little or no protection against influenza A(H3N2), although as the number of influenza A(H3N2) cases is low, the impact of this lack of protection on operational readiness was minimal. The effect of the mass vaccination programme on overall febrile respiratory infections was limited as a result of continued circulation of other pathogens of the upper respiratory tract. In spite of this, the mass influenza vaccination programme should still be recommended, particularly at the recruit camp, with vaccination carried out as soon as possible after enlistment, certainly within 14 days post-enlistment of new recruits, and ideally prior to enlistment.

## Supporting Information

**Table S1.** Summary of the number of servicemen with vaccination by time period, vaccine type and subtype, 31 May 2009 to 30 June 2012. **Table S2.** Summary of the number of servicemen with febrile respiratory illness (FRI) receiving vaccination by camp, 31 May 2009 to 30 June 2012. **Table S3.** Description of vaccines against each subtype, 31 May 2009 to 30 June 2012. **Table S4.** Number of Singapore Armed Forces (SAF) servicemen for each subtype and vaccination status by vaccine type, 31 May 2009 to 30 June 2012. **Table S5.** Crude and adjusted influenza monovalent and trivalent vaccine effectiveness against the overall influenza by camp and period, 31 May 2009 to 30 June 2012 (*n* = 7016).
