# Supplementary material for: Effectiveness of seasonal influenza vaccinations against laboratory-confirmed influenza-associated infections among Singapore military personnel in 2010–2013
Source: Influenza Other Respir Viruses. 2014 May 14;8(5):557–66. doi: 10.1111/irv.12256 (PMC4181820; doi:10.1111/irv.12256)
Supplement: Supplementary file 1 [file irv0008-0557-SD1.docx]

**Supplementary materials**

| **Time Period** | **Vaccine Type** | **Number of cases of** | | | |
| --- | --- | --- | --- | --- | --- |
|  |  | **FRI***  **(n = 7016)**  **vaccinated (%)** | **Influenza A(H1N1)pdm09**  **(n = 513)**  **vaccinated (%)** | **Influenza A(H3N2)**  **(n = 99)**  **vaccinated (%)** | **Influenza B**  **(n = 586)**  **vaccinated (%)** |
| 31/5/09 – 30/11/09 | No vaccination | N.A. | N.A. | N.A. | N.A. |
| 1/12/09 – 31/10/10 | Pandemic H1N1 | 1415 (20.2) | 43 (8.4) | 24 (24.2) | 311 (53.1) |
| 1/11/10 – 30/9/11 | Northern Hemisphere 10/11 & Southern Hemisphere 2011 | 1560 (22.2) | 46 (9.0) | 20 (20.2) | 20 (3.4) |
| 1/10/11 – 30/6/12 | Northern Hemisphere 11/12 & Southern Hemisphere 2012 | 883 (12.6) | 3 (0.6) | 8 (8.1) | 36 (6.1) |
| *FRI: Febrile respiratory illness | | | | | |

**Table S1.** Summary of the number of servicemen with vaccination by time period, vaccine type and subtype, 31 May 2009 to 30 June 2012. Dates in dd/mm/yy format.

**Table S2.** Summary of the number of servicemen with febrile respiratory illness (FRI) receiving vaccination by camp, 31 May 2009 to 30 June 2012.

| **Camp** | **No. of servicemen with FRI received Monovalent vaccination (%)** | **No. of servicemen with FRI received Trivalent vaccination (%)** |
| --- | --- | --- |
| Recruit Camp (n = 5036) | 1220 (24.2) | 1970 (39.1) |
| Other camps (n= 1980) | 195 (3.9) | 619 (12.3) |

**Table S3.** Description of vaccines against each subtype, 31 May 2009 to 30 June 2012.

| **Vaccine Type** | **Influenza A(H1N1)pdm09** | **Influenza A(H3N2)** | **Influenza B** |
| --- | --- | --- | --- |
| **Northern Hemisphere 10/11** | A/California/7/2009 (H1N1)-like virus | A/Perth/16/2009 (H3N2)-like virus | B/Brisbane/60/2008-like virus |
| **Southern Hemisphere 2011** | A/California/7/2009 (H1N1)-like virus | A/Perth/16/2009 (H3N2)-like virus | B/Brisbane/60/2008-like virus |
| **Northern Hemisphere 11/12** | A/California/7/2009 (H1N1)-like virus | A/Perth/16/2009 (H3N2)-like virus | B/Brisbane/60/2008-like virus |
| **Southern Hemisphere 2012** | A/California/7/2009 (H1N1)pdm09-like virus | A/Perth/16/2009 (H3N2)-like virus | B/Brisbane/60/2008-like virus |

**Table S4.**  Number of Singapore Armed Forces (SAF) servicemen for each subtype and vaccination status by vaccine type, 31 May 2009 to 30 June 2012.

| **Period* & Influenza Subtype** | | | **Subjects with ≤ 14 days vaccination being classified as vaccination group** | | **Subjects with ≤ 14 days vaccination being classified as no vaccination group** | |
| --- | --- | --- | --- | --- | --- | --- |
|  |  |  | Vaccination | | Vaccination | |
|  |  |  | Yes | No | Yes | No |
| **Monovalent Vaccination** | | | | | | |
| 2 | A(H1N1)pdm09 | Positive | 37 | 119 | 6 | 150 |
|  |  | Negative | 1200 | 1075 | 951 | 1324 |
| **Trivalent Vaccination** | | | | | | |
| 3 | A(H1N1)pdm09 | Positive | 18 | 38 | 12 | 44 |
|  |  | Negative | 1074 | 654 | 806 | 922 |
| 4 | A(H1N1)pdm09 | Positive | 4 | 3 | 4 | 3 |
|  |  | Negative | 1293 | 165 | 1036 | 422 |
| 3 | A(H3N2) | Positive | 9 | 14 | 6 | 17 |
|  |  | Negative | 1083 | 678 | 812 | 949 |
| 4 | A(H3N2) | Positive | 16 | 4 | 13 | 7 |
|  |  | Negative | 1281 | 164 | 1027 | 418 |
| 3 | B | Positive | 8 | 34 | 4 | 38 |
|  |  | Negative | 1084 | 658 | 814 | 928 |
| 4 | B | Positive | 43 | 17 | 11 | 49 |
|  |  | Negative | 1254 | 151 | 1029 | 376 |
| 3 | Overall† | Positive | 35 | 86 | 22 | 99 |
|  |  | Negative | 1057 | 606 | 796 | 867 |
| 4 | Overall† | Positive | 63 | 24 | 28 | 59 |
|  |  | Negative | 1234 | 144 | 1012 | 366 |
| *Period 1 refers to the pre-vaccination period; period 2 refers to the period new recruits were given monovalent vaccination; period 3 refers to the period new recruits were given trivalent vaccination; and period 4 refers to the period all SAF servicemen received trivalent vaccination.  †The overall influenza includes influenza A(H1N1)pdm09, influenza A(H3N2) and influenza B. | | | | | | |

**Table S5.** Crude and adjusted influenza monovalent and trivalent vaccine effectiveness against the overall influenza by camp and period, 31 May 2009 to 30 June 2012. (n=7016)

| **Subtype** | **Influenza Vaccine Effectiveness (%) (95% Confidence Interval)** | | | | | |
| --- | --- | --- | --- | --- | --- | --- |
|  | **Subjects with ≤ 14 days vaccination classified as vaccination group (n = 7016)** | | | **Subjects with ≤ 14 days vaccination classified as no vaccination group (n = 7016)** | | |
|  | **Crude** | **Adjusted†** | | **Crude** | **Adjusted†** | |
|  |  | **Recruit camp** | **Other camps** |  | **Recruit camp** | **Other camps** |
| **Sensitivity Analysis** | | | | | | |
| **Overall influenza, Period 3*** | 77 | 83 | 39 | 76 | 81 | 52 |
|  | (65 to 84) | (73 to 90) | (−34 to 72) | (61 to 85) | (66 to 89) | (−13 to 79) |
| **Overall influenza, Period 4*** | 69 | 77 | 34 | 83 | 93 | 47 |
|  | (49 to 81) | (59 to 87) | (−83 to 76) | (73 to 89) | (86 to 97) | (−31 to 79) |
| **Original Analysis** | | | | | | |
| **Overall influenza** | 83 | 81 | 13 | 85 | 88 | 8 |
|  | (79 to 86) | (74 to 86) | (−20 to 37) | (82 to 88) | (82 to 92) | (−27 to 34) |
| *Period 1 refers to the pre-vaccination period; period 2 refers to the period new recruits were given monovalent vaccination; period 3 refers to the period new recruits were given trivalent vaccination; and period 4 refers to the period all SAF servicemen received trivalent vaccination.  †Variables being adjusted in all logistic regression models are camp group, vaccination period, vaccination history and interaction between vaccination history and camp group. | | | | | | |
